# Neutrophils in malaria: A double-edged sword role

**DOI:** 10.3389/fimmu.2022.922377

**Published:** 2022-07-28

**Authors:** Kehinde Adebayo Babatunde, Oluwadamilola Fatimat Adenuga

**Affiliations:** ^1^Department of Physiology and Pharmacology, University of Calgary, Calgary, AB, Canada; ^2^Department of Pathology & Laboratory Medicine, University of Wisconsin, Madison, WI, United States; ^3^Department of Pathobiological Sciences, University of Wisconsin-Madison, Madison, WI, United States

**Keywords:** neutrophil, malaria, *plasmodium*, *salmonella typhimurium*, neutrophil extracellular traps (NETs)

## Abstract

Neutrophils are the most abundant leukocytes in human peripheral blood. They form the first line of defense against invading foreign pathogens and might play a crucial role in malaria. According to World Health Organization (WHO), malaria is a globally significant disease caused by protozoan parasites from the *Plasmodium* genus, and it’s responsible for 627,000 deaths in 2020. Neutrophils participate in the defense response against the malaria parasite *via* phagocytosis and reactive oxygen species (ROS) production. Neutrophils might also be involved in the pathogenesis of malaria by the release of toxic granules and the release of neutrophil extracellular traps (NETs). Intriguingly, malaria parasites inhibit the anti-microbial function of neutrophils, thus making malaria patients more susceptible to secondary opportunistic *Salmonella* infections. In this review, we will provide a summary of the role of neutrophils during malaria infection, some contradicting mouse model neutrophil data and neutrophil-related mechanisms involved in malaria patients’ susceptibility to bacterial infection.

## Introduction

According to the WHO, malaria is a globally significant disease caused by protozoan parasites from the *Plasmodium* genus. There are five species of the protozoan genus *Plasmodium* known to infect humans: *P. falciparum*, *P. vivax*, *P. malariae*, *P. ovale*, and *P. knowlesi*, of which *P. falciparum* is responsible for most cases of severe malaria and death (1). The parasites caused over 241 million clinical cases and 627,000 deaths in 2020; this represents about 14 million more cases in 2020 compared to 2019 and 69 000 more deaths. Sub-Saharan Africa continues to carry the heaviest malaria burden, accounting for about 95% of all malaria cases and 96% of all deaths in 2020 (2). There are three clinical presentations of malaria identified: severe or complicated, mild or uncomplicated (3) and asymptomatic (4). The host immune response to malaria infection varies depending on factors. Factors like the genetic make-up of parasite proteins, co-infections, host genetics, host ethnic background, and geographical locations (5, 6). The response starts with physical barriers, progresses to an innate immune response, and leads to more adaptive responses.

### Brief overview of the *Plasmodium* life cycle

The malaria parasites have a complex life cycle requiring a human and mosquito host (7). During the blood meal of the female Anopheles mosquito, sporozoites are transmitted into the human hosts (8). The pre-erythrocytic developmental stage is initiated when the released sporozoites migrate to the host liver. In the liver, the released sporozoites infect the hepatocytes (liver cells) in a process known as the liver stage. Within the hepatocytes the parasites grow and replicate as hepatic schizonts over a period of 10–12 days after which they are released as merozoites. The blood stage of the parasite starts when the merozoites rapidly invades the red blood cells (RBCs) in the bloodstream. During the blood stage, the merozoites replicates to produce more daughter parasites which are then released from the host cell upon parasite egress and subsequently re-invade RBCs to start a new asexual replication cycle (9). A small proportion of the malaria parasites will eventually differentiate into gametocytes to begin the sexual cycle which are subsequently taken up by mosquitos during the next blood meal (10).

### Neutrophil functions

Neutrophils are the most abundant white blood cell accounting for up to 70% of all blood leukocytes (11). They are also known as polymorphonuclear cells (PMNs) and are terminally differentiated leukocytes. Neutrophils are professional phagocytes, which use receptor mediated phagocytosis to internalize pathogens into phagolysosomes (12). Cytoplasmic granules include cathepsins, elastases, and myeloperoxidases that fuse with the phagolysosome to digest phagocytosed pathogens, in a process known as degranulation (12). Release of reactive oxygen species (ROS), produced *via* an NADPH oxidase-dependent process is a crucial bactericidal mechanism. Neutrophils can also kill extracellular pathogens by degranulation, secretion of ROS, or the release of neutrophil extracellular traps (NETs). NETs is the release of decondensed chromatin laced with granular proteins and histones to prevent the spread of pathogens (13).

Neutrophils are crucial for the body’s innate immune response (14, 15) and are involved in various disease processes, including pathogen infection (16), pulmonary diseases (17), cardiovascular diseases (18), inflammatory disorders (19) and cancer (20). They are challenging to study because they are short-lived effector cells of the innate immune system. Upon sensing infection, neutrophils are the first cells to migrate to the infection site (21). At the affected tissues, neutrophils use multiple antimicrobial functions such as engulfing foreign matter for internal digestion, reactive oxygen species (ROS) production and releasing NETs. The immune system plays a vital role in controlling the parasite’s growth (22). Clinical data has shown that the number of circulating neutrophils is high in patients with acute uncomplicated malaria (23), in contrast to circulating lymphocytes, which decrease during *P. falciparum* infections. During *Plasmodium* infection, parasites components and cytokines are produced and might activate circulating neutrophils. Activated neutrophils are equipped with several weapons to mount an immune defense against the parasite. While on the other hand, these neutrophil weapons might also be involved in the pathogenesis of severe malaria, though the underlying mechanism is still unclear.

During malaria infection, the immune system is overwhelmed resulting in immune suppression thereby making malaria patients to be at risk of developing secondary infections. One well-documented risk factor for invasive bacterial infection is *Plasmodium falciparum* malaria (24, 25). In Sub-Saharan Africa countries, bacterial infection in children is highly associated with malaria infection (26–28). In this review, we assess the literature examining the role of neutrophils during malaria infection and the neutrophil related mechanism involved in malaria patients’ susceptibility to bacterial infection.

## The role of neutrophils in response to *Plasmodium* parasite

One of the ways by which neutrophil play a role in the clearance of malaria parasites is by phagocytosis. Neutrophils express immunoglobulin (Ig) binding receptors Fcγ receptors and complement receptor 1 (CR1) and complement receptor 3 (CR3) (29). Phagocytosis of the released sporozoites during malaria infection are facilitated by the FcγR-receptors and by the presence of antibodies against the circum-sporozoite protein, one of the main surface antigens on sporozoites (30). Phagocytosis of parasite infected red blood cells (iRBC) *in vivo* has been observed in children with malaria (31) and in bone marrow aspirates which show neutrophils with internalized merozoites and trophozoites (32). The interaction between neutrophils and iRBCs is mediated by PfEMP1 on the iRBC surface and ICAM-1 expressed on neutrophils (33).

Neutrophil phagocytosis of free merozoites could be in an antibody dependent manner or *via* a complement mediated opsonization manner (23, 34) and can be enhanced in the presence of immune sera or when cytokines such as interferon gamma and tumor necrosis factor was added (35, 36). Recently, it was demonstrated that at high antibody levels, neutrophils are more effective *via* the action of FcγRIIA and FcγRIIIB (37). This may suggest that neutrophils are responsible for the phagocytosis of parasites in immune patients. In addition, neutrophil uptake of serum opsonized merozoites has been demonstrated *in vitro* and *ex vivo* (38). In contrast to complement dependent merozoites phagocytosis, phagocytosis of iRBC in neutrophil is largely dependent on the presence of IgG (39). Neutrophils phagocytose gametes *in vitro* in conditions similar to those of the mosquito gut when immune sera is present especially IgG (40). However, *ex vivo* evidence of the specific role of neutrophil phagocytosis of intra erythrocytic gametes in human is still lacking.

Neutrophils can clear pathogens by producing ROS by converting oxygen to superoxide *via* nicotinamide adenine dinucleotide phosphate oxidase (NADPH) oxidase (NOX). This superoxide is converted into hydrogen peroxide (H_2_O_2_) and hydroxyl radicals (-OH), collectively known as ROS (41). Neutrophils may also be involved in the control of parasite growth through antibody-dependent respiratory burst (ADRB) (35). Neutrophils isolated from malaria patients have been shown to exhibit higher ADRB activity *in vitro* and promote parasite clearance by inhibiting parasite growth (42). ROS related parasite inhibition occurs during the parasite intra-erythrocytic development stage (43) rather than during the merozoite stage. This was further demonstrated by Dasari et al. that ROS production from stimulated neutrophils does not inhibit merozoite growth *in vitro* (34). Though the mechanism underlying the observed impaired ROS production in neutrophils during malaria is unclear but released hemozoins (23) and digestive vacuoles (DV) (34) from iRBC have been suggested to be responsible.

### Evidence of NETs in Malaria

Neutrophil extracellular trap (NET) formation is an essential innate strategy for immobilizing and killing foreign pathogens by neutrophils. It occurs when activated neutrophils degranulate and release their antimicrobial factors into the extracellular environment. Several factors might induce NET formation during *Plasmodium* infections such as crystal uric acid is a potent inducer of NETosis (44) (*Plasmodium* cannot synthesize purines and imports hypoxanthine as a purine source (45). Upon erythrocyte rupture and release, xanthine dehydrogenase, which is normally present in the blood (46) will efficiently degrade it into uric acid and are released into circulation during malaria. *Plasmodium*-infected erythrocytes accumulate hypoxanthine, a precursor for uric acid), pro-inflammatory cytokines like TNF and IL8 increase during *Plasmodium* infections (47), H2O2 is secreted by immune cells stimulated by the malaria parasite and *Plasmodium* antigens induce NETosis *in vitro* (48). NETs may contribute to the host defense against sporozoites and merozoites (35). Studies have shown that in the peripheral blood of children with complicated and uncomplicated *P. falciparum* infections, NETs like structures are present (49, 50). The release of NETs might play a crucial role in controlling parasite dissemination, but on the other hand, it may also contribute to the development of severe complications. Few studies have reported the possible role of NETs in controlling parasite growth during malaria. For example, Kho and colleagues reported that NET formation was inversely associated with parasitemia levels in patients with asymptomatic malaria (51). Another study by Rodrigues et al. showed that Pulmozyme (active molecule: DNase 1) treatment to inhibit NETosis resulted in increased parasitemia levels in *P. berghei* infected mice and subsequently decreased survival rate (49). However, the group also reported that the same observation was not recorded when *P. chabaudi*-infected mice were treated with Pulmozyme. Interestingly, *Plasmodium* parasites express TatD-like DNases to cleave NETs. *In vivo* mouse data have shown that treatment of mice with recombinant TatD resulted in low parasitemia and ultimately increased survival rate (52). Knackstedt et al. demonstrated that NETs might be driving inflammatory pathogenesis in malaria (53). However, evidences that NETs released in response to malaria parasite and proof that NETs are present in tissues is still debatable. For example, Feintuch and colleagues reported that brain tissue sections from children with fatal cerebral malaria (CM) and associated retinopathy were stained with NET markers, neutrophil elastase, and citrullinated histones, with no evidence of NETs was observed (54). In contrast, a recent study by Knackstedt et al. examined and analyzed retinal tissue from fatal pediatric cases who had died of cerebral malaria. The authors showed the image of NETs by colocalizing citrullinated histone H3, elastase, and DAPI (53).

## The role of neutrophils in the pathogenesis of malaria

**Figure 1:**
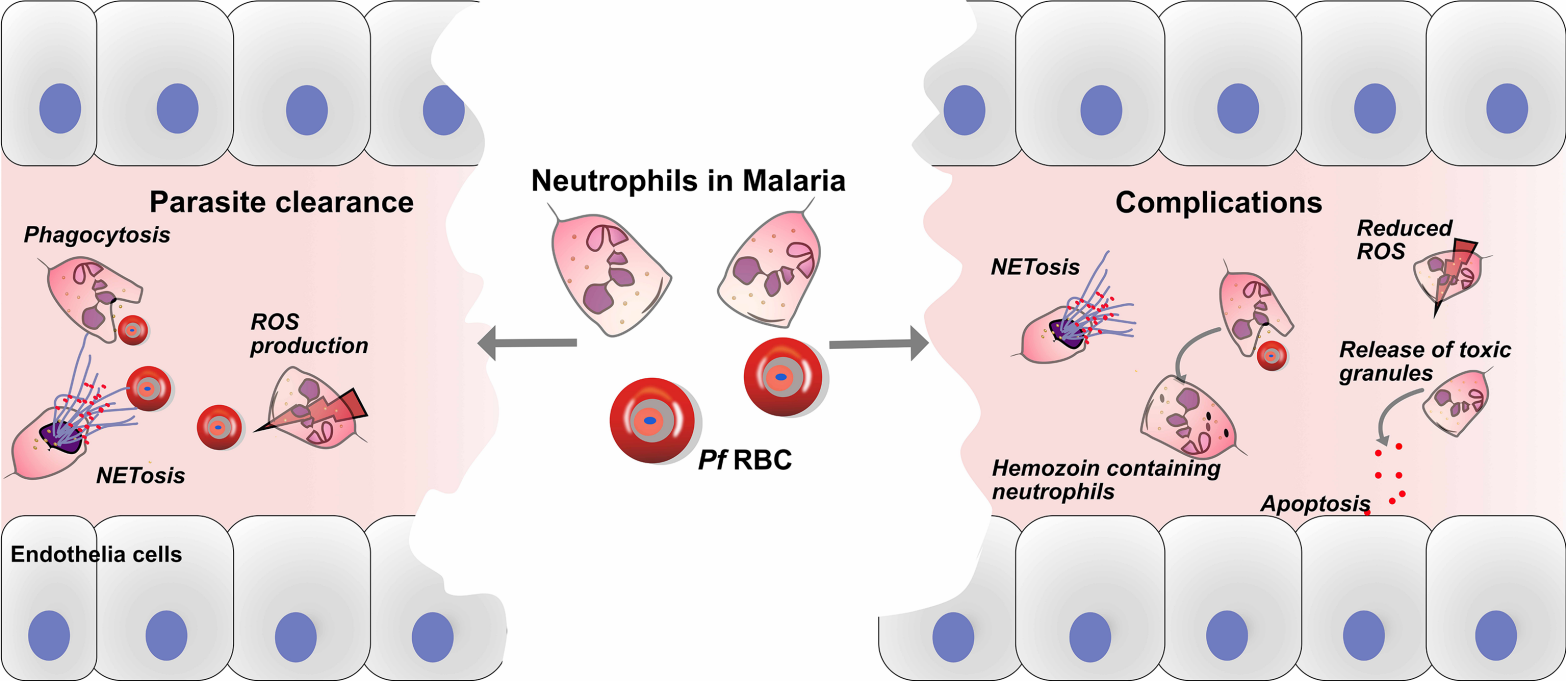
Roles of neutrophils in defense and pathology of malaria. Neutrophils play crucial roles in the immune defense against malaria, through parasite clearance *via* neutrophil phenotypic functions such as phagocytosis, reactive oxygen species (ROS) production and NETs release. On the other hand, neutrophils might play a role in the development of malaria complications, and haemozoin-containing neutrophils are associated with malaria severity. In addition, the release of toxic granules, such as myeloperoxidase (MPO), neutrophil elastase (NE) and matrix metalloproteinase-8 (MMP-8), causes endothelial cell damage *via* apoptosis. Finally, the release of NETs may aggravate complications during malaria infection.

Neutrophils may also contribute to the pathophysiology of malaria complications. Studies have associated high number of neutrophils with severe malaria cases (51, 53). The association between plasma levels of MPO, lysozyme and neutrophil lipocalin and malaria severity has been reported by several studies (35, 51, 53). In patients with severe malaria, neutrophil granule proteins such as neutrophil elastase and defensin have been observed to be increased compared to uncomplicated malaria patients (55). Another study also reported that during CM, neutrophil proteins in plasma are associated with CM and may contribute to CM pathology (endothelium damage *via* neutrophil elastase) (56) (**Figure 1**). Another indication for neutrophil activation is the release of matrix metalloproteinase (MMP)-8 and 9. For example, increased levels of plasma protein MMP-8 was reported in malaria patient, but no significant difference between uncomplicated and severe malaria. Furthermore, in Sub Saharan African children with CM, immunohistochemical staining revealed the presence of MMP-8 in the retina tissue accompanied with oedema, thus suggesting the role of MMP-8 in vascular endothelial barrier disruption (57). In addition, *in vivo* data further demonstrated that knocking out MMP-9 had no significant effect on CM development and survival in mice (58). Chemokines such as CXCLI and CXCL8 are known neutrophil chemoattractants, were at an increased level in the plasma of severe malaria patients (59). Furthermore, studies have associated a link between hemozoin laden neutrophils and disease severity has been reported in several malaria patients (60–62). In conclusion, these studies suggest a link between neutrophil activation and malaria severity.

### Murine malaria model to understand the role of neutrophils in malaria pathogenesis

Animal models have been used to study the role of neutrophils in malaria complications including lung injury, CM and liver injury (17, 63, 64). Murine models have demonstrated the association of accumulated neutrophils in the lungs with lung injury. Murine models of CM have demonstrated that neutrophils express cytokines such as IL2, IL12, IL18, IFNγ, and TNF and chemoattractive-chemokines (65) suggesting a role for neutrophils in cytokine and chemokine secretion during CM. Nacer and colleagues reported that neutrophils in murine CM are detected in the vasculature (66) and their depletion prevented CM development (67). Using chimeric mice, Ioannidis and colleagues identified neutrophils as the main cellular sources of CXCL10, a CXCR3 binding chemokine which is essential for the attraction of pathogenic CD8+ T cells to the brain in murine CM (68). In addition, studies revealed that circulating levels of CXCL10 is the most accurate predictor of CM mortality (69) (70) and its neutralization with specific mAbs, significantly prevents brain intravascular inflammation and protects infected animals from CM by reducing peripheral parasitemia level (71–73).

### Murine malaria models: Neutrophil contradicting data

Murine models have over the years played a valuable role in understanding the role of neutrophils in malaria (**Table 1**) however, there are contradicting data on the role of neutrophils using the mouse model, and as such, neutrophils’ role in malaria is still unclear. For example, Schumak et al. (74) suggested that the number of circulating neutrophils in the blood and brain increased in *P. berghei* ANKA infected mice, while in contrast, Pai et al. reported no increase in the number of circulating neutrophils in the brain (79) though the experimental approach to count neutrophils were different in the two studies. In Schumak study, brain tissue was fixed in buffered formalin and quantification of neutrophils in tissue sections was performed in 10 high power fields (HPF). While in Pai study, neutrophils were quantified in real time using 2-photon intravital microscopy. Another contradiction is the link between neutrophil depletion and survival rate in murine CM model. For example, some *in vivo* studies demonstrated that depletion of neutrophils using anti-CD11a resulted in an increased survival rate and slowed down the rate of CM development (67, 80) (**Table 1**). In these studies, depletion of neutrophils was done using anti-CD11A or anti-GR1 before parasite infection, which depleted neutrophils and other leukocytes. In contrast, when anti-GR1 was administered late during the infection, the authors still observed CM development in their model except for a study by Senaldi and colleague (76). In contrast, another study showed that when specific anti-Ly6G antibody was used for neutrophil depletion, no CM development was observed (74) (**Table 1**). Interestingly, neutrophil released CXCL10 is known to recruit pathogenic CD8^+^ T cells to the brain in murine CM (68). The indirect effect on CD8+ T cells in the murine CM by the anti-GR1 antibody might explain the contradicting data. Therefore, there is a need for more in depth studies to elucidate whether neutrophils count plays a role in the development of CM.

**Table 1:** Showing studies on neutrophil in murine malaria.

Mouse strain	Parasite strain/Load/route of infection	Procedure for neutrophil depletion	Strategy of neutrophil detection	Outcome of neutrophil depletion	Ref.
C57BL/6	Transgenic *P.berghei* ANKA expressing ovalbumin (PbTg) infected RBC/5*10^4/^i.v	250 μg of anti-GR1(day 0 of infection or day 3-5 p.i for 30 mins i. p^1^)	Flow cytometry on blood samples: CD11b^+^ Ly6C^int^ Ly6G^+^	CM developed on day 6 post infection, survival rate was 80% and no effect on parasitemia level.	(74)
C57BL/6	Transgenic *P.berghei* ANKA expressing ovalbumin (PbTg) infected RBC/5*10^4/^i.v.	250 μg of anti-GR1(day 3 and day 5 i.p)	Flow cytometry on blood samples: CD45+ Ly6C^int^ Ly6G+	Decreased CM development on day 6 p.i, high survival rate and no effect on parasitemia level.	(74)
C57BL/6	*P.berghei* -ANKA infected RBC/1*10^6^/i.p.	200 μg of anti-CXCL10 (between day 3 and 9 p.i)	Flow cytometry on spleen samples: Ly6G+	High CM, low survival rate and low parasitemia level.	(68)
C57BL/6	*P.berghei* -ANKA infected RBC/1*10^5^/i.v.	Pulmozyme (5mg/kg for every 8h i.p.)	Not Applicable	Low survival rate at 20%, 100% mortality by day 10 post infection and parasitemia increased day 6 p.i.	(49)
C57BL/6, BALB/c	*P.berghei* -ANKA and NK65 infected RBC/1*10^6^/i.p.	Immunization with r*Pb*TatD or rPcTatD (50 μg intra-muscularly every 14 days).	Not Applicable	100% survival rate and reduced parasitemia level.	(52)
CBA/NSlc	*P.berghei* -ANKA infected RBC/1*10^6^/i.p.	250 μg of anti-GR1 (day 1 or day 5 i.p.)	Tail blood	no CM development (low hemorrhage), survival rate was 90% by day 10 post infection and no effect on parasitemia level.	(67)
DBA/2	*P.berghei* -ANKA infected RBC/1*10^6^/NA	anti-GR1(0.2mg on day 1 p.i)	Flow cytometry on blood samples and microscopy of blood smear	Low MA-ARDS development, survival rate was 90% by day 10 post infection and no effect on parasitemia level.	(17)
129/Ola + C57BL/6J mice	*P.berghei* -ANKA infected RBC/1*10^7^/NA	anti-GR1(1mg on day 6 post infection i.p.)	Flow cytometry on blood samples: GR1+	There was 60%-80% of CM development and survival rate was 0% after 24h of CM development.	(75)
CBA/Ca	*P.berghei* -ANKA infected RBC/1*10^6^/i.p.	anti-GR1(0.5mg on day 5 post infection i.p.)	Microscopy of blood smear	BBB was not disrupted, delayed death and high survival rate (80%).	(76)
BALB/c	PyMDR/NA	anti-GR1(day 3 until day 7 post infection i.p.)	Not Applicable	Low liver injury (low AST, ALT and ALP levels^2^).	(77)
C57BL/6	*P.chabaudi* AS infected RBC/1*10^4^/i.v.	DNase^-/-^	Flow cytometry on blood samples: CD45+ CD3− Ly6G/Chi	Low liver injury, low AST level and no effect on parasitemia level.	(53)
C57BL/6	*P.chabaudi* AS infected RBC/1*10^4^/i.v.	NE/PR3−/−	Flow Cytometry on blood samples: CD45+ CD3− Ly6G/Chi	Low liver injury, low AST level and no effect on parasitemia level.	(53)
C57BL/6	*P.chabaudi* AS infected RBC/1*10^4^/i.v.	Anti-G-CSF (day 7 post infection i.v.)	Flow Cytometry on blood samples: CD45+ CD3− Ly6G/Chi	Low liver injury, low AST level.	(53)
C57BL/6	*P. yoelli* 17XNL infected RBC/2*10^4^/i.v.	MPO−/−	Not Applicable	Not determine, however no effect on parasitemia level on day 6-12 post infection, parasitemia level increased after day 12 post infection.	(78)
C57BL/6 + *Py*17XNL	*P. yoelli* 17XNL infected RBC/2*10^4^/i.v.	Anti-Ly6G (500 μg i.p.)	Flow Cytometry on blood samples: CD11b+ Ly6Cint GR1+	Not determine, however no effect on parasitemia level.	(78)

i.p., Intraperitoneal; i.v., Intravenously; p.i, Post infection; i.m., Intramuscularly; AST, aspartate aminotransferase; ALT, alanine aminotransferase; ALP, alkaline phosphatase.

## Malaria co-infection with bacteria: Any neutrophil link?

*Salmonella* co-infection is a common bacterial infection, and it remains a global health concern. One well-documented risk factor for *Salmonella* is *Plasmodium falciparum* malaria (25). It’s often a lethal complication of *P. falciparum* infection in Sub-Saharan Africa. In Gambia, the incidence of invasive NTS infection mirrors that of malaria, and about 43% of children with *Salmonella* bacteremia had concurrent *P. falciparum* infections (81). In Tanzania, invasive NTS in young children is highly associated with recent malaria infection (26). Interestingly evidence from co-infection models supports this idea (**Table 2**). For example, a study by Cunnington and colleagues demonstrated that prior infection of mice with non-lethal *P. yoelii* resulted in decreased survival of *S. typhimurium* in the mice. Though the authors suggested that the decrease in the survival rate of the mice was a result of impaired ROS production in neutrophils (85), the mechanism underlying the neutrophil-associated immune suppression remains unclear.

**Table 2:** Showing studies on malaria and salmonella co-infection.

*Plasmodium *spp.	Bacteria Strain	Bacteria load	Route of bacteria challenge	Time of bacteria Co infection	End point	Bacteria-related Outcome	Animal strain	Ref.
*P. yoelii* *nigeriensis*	*S.* typhimurium strain (IR715)	1×10^8^ CFU^3^.	Intragastric	Day 10	Day 14	Reduced intestinal inflammation to NTS	CBA/J	(82)
*P. yoelii* *nigeriensis*	*S.* typhimurium strain (IR715)	Not available	Intragastric	Day 10	Day 11	Increased NTS colonization in feces.	C57BL/6J	(83)
*P. yoelii* *nigeriensis*	*S.* typhimurium strain (IR715)	1×10^8^ CFU.	Intra- peritoneal	Day 10	Day 12, 13	Increased CFU in liver.	CBA/J	(84)
*P. fragile*	*S.* typhimurium strain (IR715)	1×10^8^ CFU.	Ligated ilieal loops	Day 14, 15	8h	Reduced intestinal inflammation to NTS.	*Macaca mulatta*	(82)
*P. yoelii* *nigeriensis*	*S.* typhimurium strain (IR715)	1×10^8^ CFU.	Intragastric	Day 0	Day 5	Increased CFU in liver, spleen and peyer’s patch and spleen	CBA/J	(84)
*P. yoelii* 17XNL	12023-GFP	1x10^5^ CFU	Intra- peritoneal	Day 15	18h	Increased CFU in blood, spleen, and liver.	C57BL/6	(85)
*P. yoelii* 17XNL	*S.* typhimurium strain (BRD509)	1×10^8^ CFU.	Intravenous	Day 14, 28	Day 17, 31	Increased CFU in liver	C57BL/6	(86)

CFU, Colony Forming Unit.

### Underlying mechanisms of neutrophil associated immune suppression during malaria

During malaria infection, the parasite continuously breaks down RBCs followed by eryptosis of many uninfected RBCs (87). The direct destruction of RBC leads to the release of hemoglobin, or its breakdown product heme, into the plasma. Free heme is prooxidant and highly cytotoxic, contributing to endothelial injury (88). Intracellular heme is then degraded into equimolar amounts of iron, carbon monoxide, and biliverdin through the action of heme oxygenase (HO). Significantly, plasma heme is raised during both acute (89) and subclinical (90) *P. falciparum* malaria infections in humans and during acute *P. yoelii* infection in mice (85) https://jlb.onlinelibrary.wiley.com/doi/full/10.1002/JLB.3RI1018-400R - jlb10293-bib-0063. Studies have shown that hemolysis might be responsible for inhibiting neutrophil functions during malaria. For example, Cunnington and colleagues using a malaria mouse model demonstrated that neutrophils from malaria-infected mice could phagocytose S. typhimurium; however, their ability to kill was impaired.

The authors further reported that the phagocytosed bacteria remained viable and replicated within the phagosome due to deficient ROS production (85). In another study, neutrophils from children with acute malaria were observed to exhibit impaired ROS production. The authors recorded that the dysfunctional ROS production persists for up to 8 weeks after drug treatment (89). The authors suggested that this might explain why children with acute malaria remain susceptible to secondary bacterial infection (27). Several studies have shown that during malaria infection, the migration of neutrophils into the infected tissues, including blood (85), intestine (82), and liver (84) is impaired. Neutrophils precursors of *Plasmodium*-infected mice have been shown to express heme oxygenase-1(HO-1) (85), which has been reported to reduce neutrophil migration into the inflamed lung (91), thus suggesting an association between HO-1 and neutrophil migration.

During the parasite life cycle in the RBC, the malaria parasite feeds on the hemoglobin and packages the waste product hemozoin in an organelle designated the digestive vacuole (DV) (92). Large numbers of DVs are released into blood circulation during severe malaria infection. Some reports have implicated the role of DVs in suppressing the host immune system *via* the inhibition of neutrophil functions. For example, a study by Dasari and colleagues demonstrated that phagocytosed DVs induce oxidative burst in human neutrophils. However, the capacity to generate a subsequent ROS response to kill phagocytose bacteria was impaired (34). Thus, the anti-microbicidal activity was compromised. They suggested that DV might explain the risk of developing bacterial sepsis in patients with severe malaria. However, much more work is needed to fully characterize the content of these DVs and their effect on neutrophil migration during malaria infection.

## Future direction and conclusion

Neutrophils in malaria remain understudied and they play a double-edge sword role in malaria. During malaria infection, neutrophils may be involved in defense mechanisms against parasites *via* phagocytosis and ROS production. On the other hand, neutrophils might also be involved in the pathogenesis of severe malaria *via* NETs and toxic granule proteins release. Therefore, more research studies are required to understand the specific roles of neutrophils in malaria.

During malaria, small extracellular vesicles (EVs) are secreted by *Plasmodium* infected RBC (iRBCs). EVs contribute to the immune regulation by transferring cargoes including RNAs from the iRBCs to immune cells, resulting in immune suppression or immune activation depending on the cellular context. Currently, it’s not clear if these EVs are involved in the observed immune suppression *via* the modulation of neutrophil functions in malaria patients. It will be interesting to know if EVs can deliver their biological cargoes to neutrophils which in turn maybe responsible for the observed immune suppression.

There seems to be a lot of contradicting neutrophil data using both *in vitro* and *in vivo* malaria models. We suggest that a novel *ex vivo* microfluidic platform, modelling the *in vivo* malaria infection microenvironment, will allow studying the interaction of neutrophils and malaria at single cell resolution and in real time. Such platform should allow the investigation of neutrophil migration, NETs release and parasite killing during neutrophil-parasite interaction.

## Author contributions

KB conceptualized the review. KB and OA drafted the review. KB prepared the figure and we read and adapted the manuscript and approved the final version.

## Funding

This work is supported by the Swiss National funding (P500PB_203002) and a grant from the Jubilee Foundation of the Swiss Life Insurance and Pension Fund for Public Health and Medical Research to KB.

## Conflict of interest

The authors declare that the research was conducted in the absence of any commercial or financial relationships that could be construed as a potential conflict of interest.

## Publisher’s note

All claims expressed in this article are solely those of the authors and do not necessarily represent those of their affiliated organizations, or those of the publisher, the editors and the reviewers. Any product that may be evaluated in this article, or claim that may be made by its manufacturer, is not guaranteed or endorsed by the publisher.

## Footnotes

1 Abbreviations: i.p., Intraperitoneal; i.v., Intravenously; p.i, Post infection; i.m., Intramuscularly

2 Abbreviations: AST, aspartate aminotransferase; ALT, alanine aminotransferase; ALP, alkaline phosphatase.

3 Abbreviation: CFU, Colony Forming Unit.
